# Insulin-like growth factor binding protein-3 is a new predictor of radiosensitivity on esophageal squamous cell carcinoma

**DOI:** 10.1038/srep17336

**Published:** 2015-12-16

**Authors:** Li-Ling Luo, Lei Zhao, Ying-Xue Wang, Xiao-Peng Tian, Mian Xi, Jing-Xian Shen, Li-Ru He, Qiao-Qiao Li, Shi-Liang Liu, Peng Zhang, Dan Xie, Meng-Zhong Liu

**Affiliations:** 1 Department of Radiation Oncology, Sun Yat-Sen University Cancer Center, Guangzhou, China; 2 State Key Laboratory of Oncology in South China, Sun Yat-Sen University Cancer Center, Guangzhou, China; 3 Department of Hepatobiliary Surgery, Sun Yat-sen Memorial Hospital, Sun Yat-sen University, Guangzhou, China; 4 Medical Imaging and Interventional Center, Sun Yat-Sen University Cancer Center, Guangzhou, China; 5 Collaborative Innovation Center for Cancer Medicine, Guangzhou, 510060, China

## Abstract

Insulin-like growth factor binding protein-3 (IGFBP-3) plays an essential role in radiosensitivity of esophageal squamous cell carcinoma (ESCC). However, the underlying mechanism is not completely understood. Here, we observed that IGFBP-3 had favorable impact on the tumorigenicity of ESCC cells in nude mice by using an *in vivo* imaging system (IVIS) to monitor tumor growth treated with ionizing radiation (IR). Downregulation of IGFBP-3 expression enhanced tumor growth, inhibited anti-proliferative and apoptotic activity and result in IR resistance *in vivo*. Cell cycle antibody array suggested that silencing IGFBP-3 promoted transition from G0/G1 to S phase, perhaps though influencing Smad3 dephosphorylation and retinoblastoma protein (Rb) phosphorylation. Downregulation of P21 and P27, and upregulation of p-P27 (phospho-Thr187), cyclin-dependent kinase 2 (CDK2), and cyclin E1 might contribute to the G0/G1 to S phase transition promoted by IGFBP-3. Our results suggest that Smad3-P27/P21-cyclin E1/CDK2-phosphorylated retinoblastoma protein pathways might be involved in this IGFBP-3 mediated radiosensitivity transition in ESCC.

Esophageal cancer (EC) is the sixth leading cause of cancer mortality worldwide^1^. The most prevalent histologic type of EC in China and eastern countries is esophageal squamous cell carcinoma (ESCC)^2^. Neoadjuvant chemoradiotheray or chemotherapy followed by surgery and definitive chemoradiotherapy are the standard regimen worldwide^3^. The 5-y survival rates of ESCC by these therapies reported are 30–65%^4^,^5^. However, the susceptibility of individual tumors varies widely, with some patients showing no response to chemoradiotherapy (CRT) or experiencing adverse effects. Local failure of radiotherapy remains a major concern, and persistent or recurrent disease is reported in 40–60% of treated patients^6^. To date, several previous studies have reported gene expression profiles for CRT sensitivity in ESCC^7^,^8^,^9^. Thus, it would be desirable to identify the mechanism of ESCC radiosensitivity and the key molecules involved.

Insulin-like growth factor binding protein-3 (IGFBP-3) is one of the six secretory glycoproteins of the IGFBP family and is a major regulator of insulin-like growth factor (IGF)-1 and -2^10^,^11^. Over the past decade it has become widely appreciated that IGFBP-3 contributes to the pathophysiology of various human diseases, including cancer, diabetes, and malnutrition^12^,^13^,^14^. Previous studies have suggested that IGFBP-3 interacts with various proteins or signaling cascades crucial to cell cycle control. For example, IGFBP-3 plays a role in the cell cycle progression of Myc prostatic epithelial cells^15^ and induced G1 cell cycle arrest with inhibition of cyclin-dependent kinase (CDK) 2 and 4 in MCF-7 human breast cancer cell^16^. These findings suggest that IGFBP-3 not only has an antiproliferative effect through inducing apoptosis but also regulates the cell cycle. However, the exact mechanisms underlying the induction of cell cycle arrest by IGFBP-3 remain to be determined.

A recent study showed that nimotuzumab, an anti-epidermal growth factor receptor (EGFR) monoclonal antibody that binds to the extracellular domain of the EGFR and inhibits epidermal growth factors binding, increases ESCC radiosensitivity dependent on the upregulation of IGFBP-3^17^. Furthermore, reduced IGFBP-3 expression may be a risk factor for advanced clinicopathologic classification and poor patient survival and could serve as a prognostic marker for the evaluation of ESCC patients^18^. Given these findings and the biological importance of IGFBP-3, we sought to determine the role of IGFBP-3 in ESCC radiosensitivity and its potential mechanisms.

## Results

### IGFBP-3 expression influenced cell viability under IR *in vitro*

**Figure 1:**
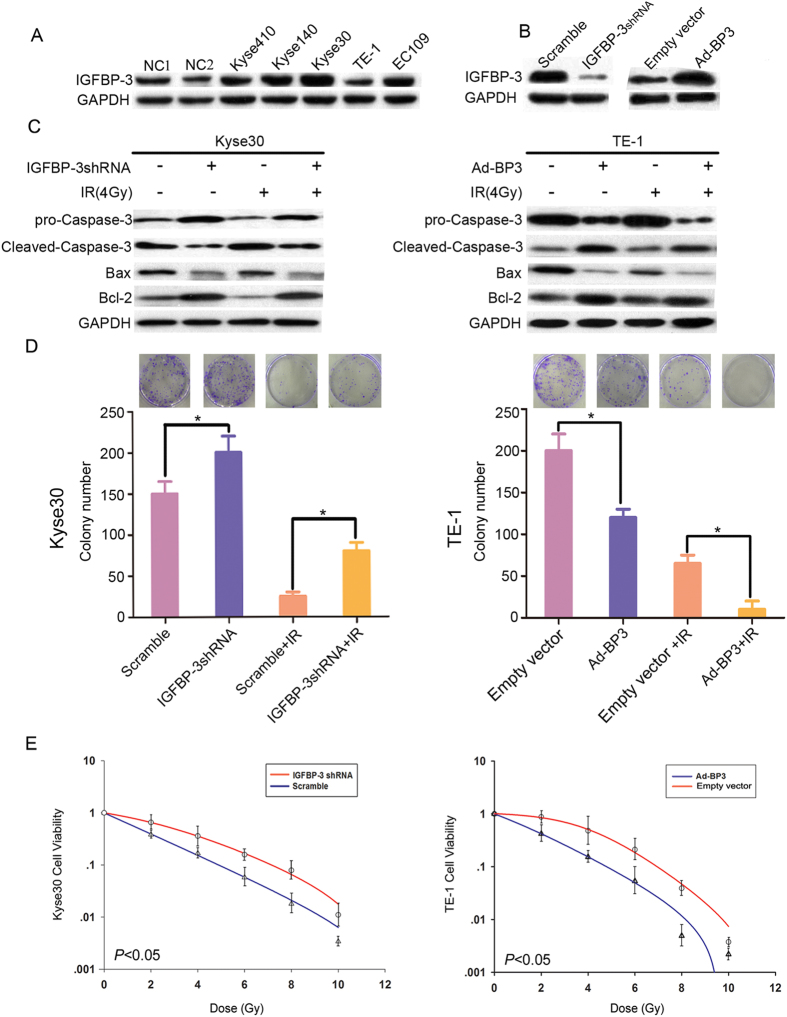
IGFBP-3 expression in human esophageal squamous cell carcinoma (ESCC) cells and confers resistance to IR on esophageal squamous cell carcinoma (ESCC) cells *in vitro*. (**A**) Western blot analysis shows the expression of IGFBP-3 in five ESCC cell lines (Kyse410, Kyse140, Kyse30, and TE-1) and non-neoplastic esophageal control tissues (NC1 and NC2). (**B**) IGFBP-3 shRNA was introduced into Kyse30, for stable knockdown of IGFBP-3 through recombinant lentiviral infection. Overexpression of IGFBP-3 was induced into TE-1 by an adenoviral vector (Ad-BP3). Empty vector was used as a control. IGFBP-3 levels in these two cell lines were examined by western blotting. GAPDH was used as a loading control. (**C**) The relationship between IGFBP-3 expression and apoptosis in ESCC cell lines under IR was examined by western blot analysis. (**D**) The effect of IR (4Gy) on colony formation by IGFBP-3 shRNA transfected Kyse 30 cells and Ad-IGFBP3 infected TE-1 cells was determined. Data represent the mean ± standard error of three independent experiments. ^*^Compared with control group (*P* < 0.05). (**E**) Radiation cell survival curves of IGFBP-3 shRNA transfected Kyse 30 cells and Ad-IGFBP-3 infected TE-1 cells. Data represent the mean ± standard error of three independent experiments. The clonogenic cell survival assay was described in Materials and methods.

Expression of IGFBP-3 in five ESCC cell lines was analyzed by western blotting (Fig. 1A). Results revealed that IGFBP-3 protein was highly expressed in the Kyse 30 cells, whereas expression low in TE-1 cells, respectively. IGFBP-3-specific shRNA was used to knock down the IGFBP-3 gene in the Kyse30 cells. Overexpression of IGFBP-3 was induced into TE-1 cells by an adenoviral vector (Ad-IGFBP-3). Empty vector (EV) was used as a control (Fig. 1B). We found that after IGFBP-3 knockdown, Kyse30 cells displayed a substantial drop expression of IGFBP-3 compared with Kyse 30-vector cells. However, after infected into Ad-IGFBP-3, TE-1 cells showed a significant increase in the expression of IGFBP-3 compared with the TE-1-vector cells. Next, we investigated the effects of IGFBP-3 on ESCC cell response to IR *in vitro*. Kyse30 and TE-1 cell viability before and after IR exposure was then measured by MTT proliferation assay (Supplementary Figure 1). Cell survival under IR was substantially decreased in Kyse 30-vector cells and TE-1 Ad-IGFBP-3 cells, however, increased in Kyse 30-shRNA cells and TE-1-vector cells.

### Knockdown of IGFBP-3 confers resistance to IR on ESCC cells *in vitro*

We next assessed the relationship between IGFBP-3 expression and apoptosis in ESCC cell lines under IR. We examined caspase-3 activity and levels of Bcl-2/Bax protein under IR in ESCC cells. We observed that apoptosis related caspase-3 activity was significantly reduced in Kyse-IGFBP-3shRNA cells and TE-1-vector cells. Upregulation of Bcl-2 and downregulation of Bax protein expression was observed in them despite exposure to IR. On the contrary, upregulation of IGFBP-3 expression enhanced apoptosis in Kyse30-vector cells and TE-1-Ad-IGFBP3 cells (Fig. 1C). A colony formation assay was used to determine how ESCC cell proliferation responds to IR. In Kyse30-IGFBP-3 shRNA cells, the number of colonies increased compared with control cells after IR. TE-1-Ad-IGFBP-3 cells led to smaller colony numbers compared with control cells after IR (Fig. 1D,E). These results demonstrate that downregulation of IGFBP-3 enhanced cell viability under IR whereas overexpression of IGFBP-3 accounted for radiosensitivity in ESCC cells line.

### IGFBP-3 arrests the cell cycle in G1

**Figure 2:**
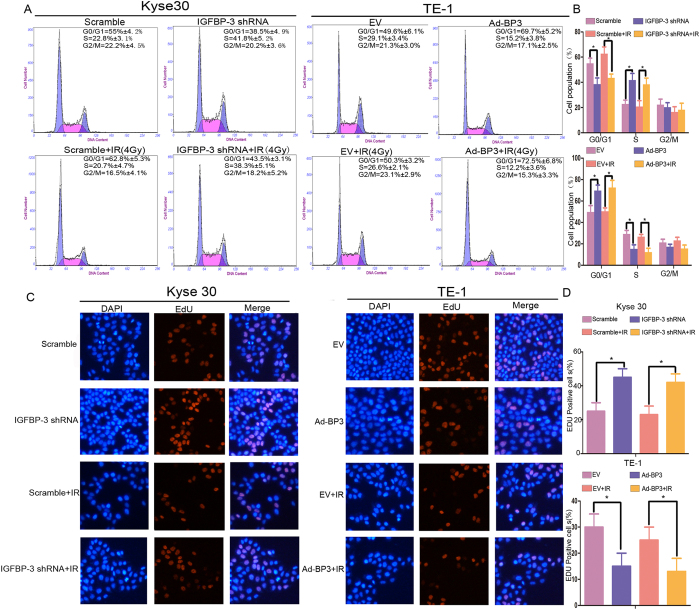
IGFBP-3 promoted G1 cell cycle arrest in esophageal squamous cell carcinoma (ESCC) cells. (**A**) Histograms show the percentages of cells in the G1, G2, and S phases under IR (4Gy), determined by flow cytometry. (**B**) The percentage of cell cycle distribution was shown as the mean ± S.D. from three independent experiments. ^*^Compared with control group (*P* < 0.05). (**C**) Kyse30 and TE-1 cells were subjected to IR (4Gy) followed by labeling with 5-ethynyl-2-deoxyuridine (EdU) for 3 h. (**D**) EdU positive cells were quantified using Image J software. Data represent the mean ± standard error of three independent experiments with triplicate wells.^*^Compared with control group (*P* < 0.05).

To identify potential mechanisms for the modulation of ESCC IR resistance by IGFBP-3, we investigated whether IGFBP-3 induced cell cycle arrest by flow cytometry and EdU assay. Changes in cell cycle phase distribution based on the expression of IGFBP-3 were determined by flow cytometry. As shown in Fig. 2A and B, before IR resulted in a significant decrease in the percentage of cells in G0/G1 from 55% to 38.5% and an increase in the percentage of cells in S phase from 22.8% to 41.8% in Kyse30-Vector cells line, whereas from 62.8% to 43.5 and 20.7% to 38.3% after IR in Kyse 30 IGFBP-3-shRNA cells. Similarly, the percentage of cells in G0/G1 increased from 49.6% to 69.7% and the percentage of cells in S phase from 29.1% to19.2% in TE-1-vector control cells, whereas from 50.3% to 72.5% and 26.6% to12.2% in TE-1-Ad-IGFBP-3 cells. There were no significant changes in the number in G2/M. An EdU assay was used to detect DNA synthesis. As shown in Fig. 2C and D, EdU positive cells were increased among Kyse30-IGFBP-3shRNA cells compared with controls regardless of IR. Furthermore, overexpression of IGFBP-3 in TE-1-Ad-IGFBP3 cells markedly decreased EdU positive cell numbers compared with controls regardless of IR. These findings suggest that knockdown of IGFBP-3 promotes cell cycle transition from G0/G1 to S phase and upregulation of. IGFBP-3 results in G1/S arrest.

### Expression of IGFBP-3 affects the therapeutic effect of IR on ESCC cell xenografts

**Figure 3:**
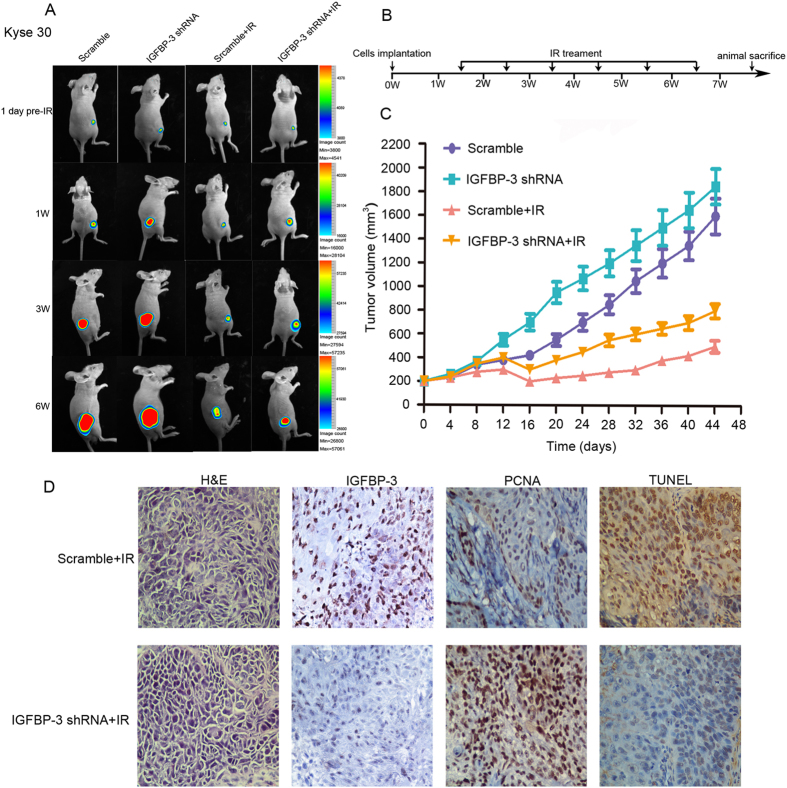
Silence of IGFBP-3 inhibits the therapeutic effect of IR on ESCC cell xenografts. Kyse30-IGFBP-3shRNA and Kyse30-vector cells (3 × 10^6^) were injected s.c. into the lower limbs of athymic nude mice, respectively. When the volume of a transplanted tumor reached 200 mm^3^, mice were treated with a 6 Gy dose of IR. (**A**) Bioluminescent images of ESCC tumor xenografts was applied to detected the tumor size on 1day before IR treatment, 1 week, 3 weeks and 6 week after implantation of ESCC cells. (**B**) The procedure chart was shown. (**C**) The tumor volume of xenografts was measured with calipers every 3 days for a total of 35–44 days .The values represent mean tumor volume ± SE. (**D**) Representative images showing xenograft tumors in null mice from control and Kyse30-IGFBP-3 shRNA cells after IR treatment. H&E staining, IHC staining of IGFBP-3 and PCNA, and TUNEL assays were performed.

**Figure 4:**
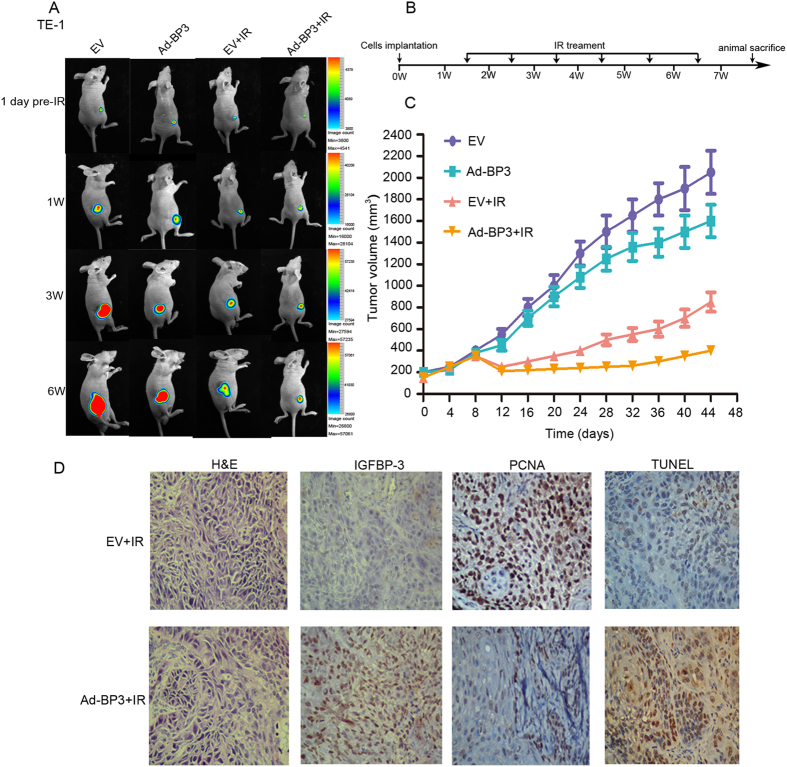
Overexpression of IGFBP-3 enhances the therapeutic effect of IR on ESCC cell xenografts. TE-1 cells infected with EV or Ad-IGFBP-3 (3 × 10^6^) were injected s.c. into the lower limbs of athymic nude mice, respectively. When the volume of a transplanted tumor reached 200 mm^3^, mice were treated with a 6 Gy dose of IR. (**A**) Bioluminescent images of ESCC tumor xenografts was applied to detected the tumor size on 1day before IR treatment, 1 week, 3 weeks and 6 week after implantation of ESCC cells. (**B**) The procedure chart was shown. (**C**) The tumor volume of xenografts was measured with calipers every 3 days for a total of 35-44 days .The values represent mean tumor volume ± SE. (**D**) Representative images showing xenograft tumors in null mice from control and Kyse30-IGFBP-3 shRNA cells after IR treatment. H&E staining, IHC staining of IGFBP-3 and PCNA, and TUNEL assays were performed.

We conformed if the expression of IGFBP-3 could affect the response of ESCC cells to IR *in vivo*. Kyse 30-IGFBP-3shRNA-Luc2 and Kyse-vector-Luc2 cells were inoculated into subcutaneously into the legs of athymic nude mice and used an *in vivo* imaging system (IVIS) to monitor tumor growth in mice treated with IR. As shown in Fig 3A,B, we observed that knockdown of IGFBP-3 expression enhanced ESCC cell tumorigenicity, and the ESCC xenografts grew more rapidly in null mice compared with control. Furthermore, the growth of the Kyse30-IGFBP-3 shRNA tumors after IR significantly increased (from a mean tumors volume of 200 mm^3^ before IR treatment, adding to 780 ± 80.5 mm^3^ at the end of treatment), compared with the control (from a mean tumors volume of 200 mm^3^ to 400 ± 40.7 mm^3^, before and after the same treatment) (*P* < 0.05; Fig. 3C). The cell proliferation was observed on a rise in Kyse30-IGFBP-3 shRNA xenograft groups (mean LI of PCNA = 80.1 ± 3.6%) compared with the control (mean labeling index, LI, of PCNA = 35 ± 3.1%, *P* < 0.05). However, decreased cell apoptosis was demonstrated in the Kyse30-IGFBP-3 shRNA xenograft group (mean apoptotic index, AI = 20 ± 4.5%) compared with that in control (mean AI = 85 ± 10.2%, *P* < 0.05). Similarly, after IR, tumors formed from TE-1-Ad-IGFBP-3-Lus2 cells (from a mean tumor volume of 200 mm^3^ to 250 ± 30.7 mm^3^, before and after the same treatment) implanted in nude mice grew more slowly a than those formed by the control (from a mean tumor volume of 200 mm^3^ to 550 ± 49.7 mm^3^, before and after the same treatment, *P* < 0.05, Fig. 4A,C). In addition, cell proliferation was found decrease in TE-1-Ad-IGFBP-3-Lus2 cells xenograft groups (mean LI of PCNA = 25.1 ± 5.6%) compared with the control (mean labelling index, LI, of PCNA = 70 ± 8.1%, *P* < 0.05). However, increased cell apoptosis was demonstrated in the TE-1-Ad-IGFBP-3-Lus2 cells xenograft group (mean apoptotic index, AI = 80 ± 6.5%) compared with that in the control (mean AI = 30 ± 11.6%, *P* < 0.05). In addition, IHC staining of PCNA (a widely used cell proliferation marker) and TUNEL assay for cell apoptosis were performed on sections of these xenograft tumors in IR treatment (Figs 3D and 4D). These results revealed that inhibition of IGFBP-3 suppressed the ESCC killing effect of IR and upregulation of IGFBP-3 expression improve radiosensitivity *in vivo*.

### *Expression of IGFBP-3* regulated the expression and phosphorylation of key cell cycle proteins

**Figure 5:**
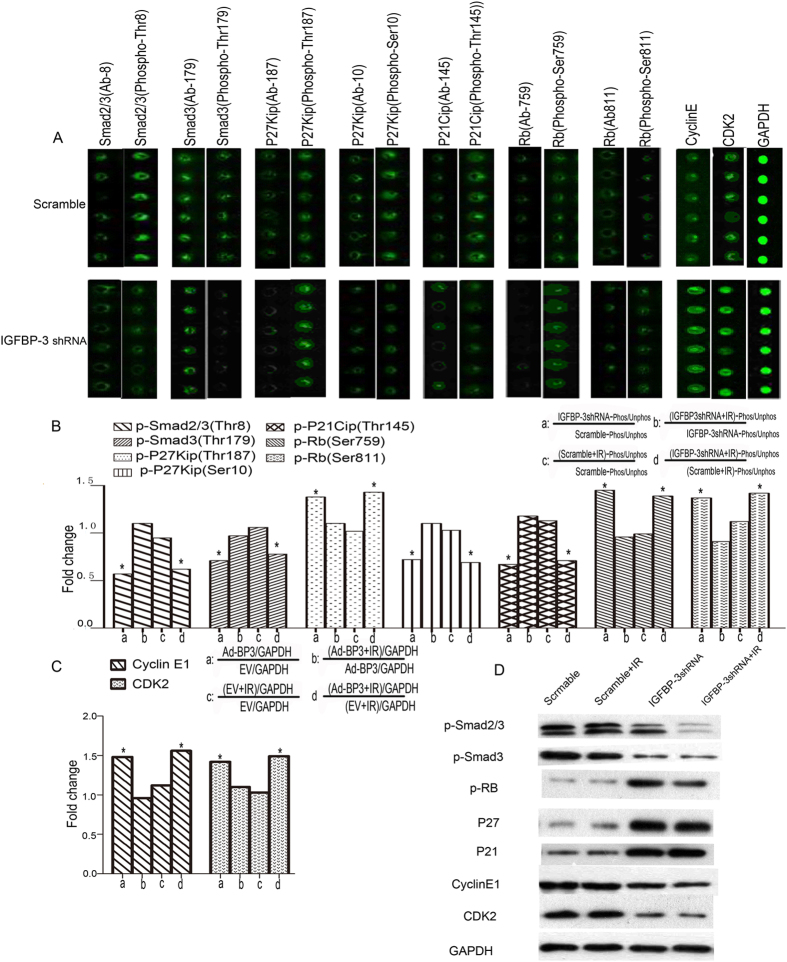
Silencing endogenous IGFBP-3 regulated the expression and phosphorylation of key cell cycle proteins. (**A**) Kyse30 cells transfected with either IGFBP-3 shRNA or an empty vector as a control were treated with or without IR (4 Gy) respectively and were lysed and subjected to an antibody microarray. (**B,C**) Portions of the array illustrate differential expression of cyclin E1 and cyclin-dependent kinase (CDK2) and phosphorylation of Smad2/3, Smad3, and retinoblastoma protein (Rb) in control cells, IGFBP-3-silenced Kyse30 cells, control cells after IR treatment and IGFBP-3-silenced Kyse30 cells after IR treatment .The fold change in cell cycle related proteins was measured. Each panel contains six replicates of a specific antibody–protein reaction. ^*^Compared with control group (*P* < 0.05). (**D**) The expression and phosphorylation of key cell cycle proteins were demonstrated by western blot analysis.

**Figure 6:**
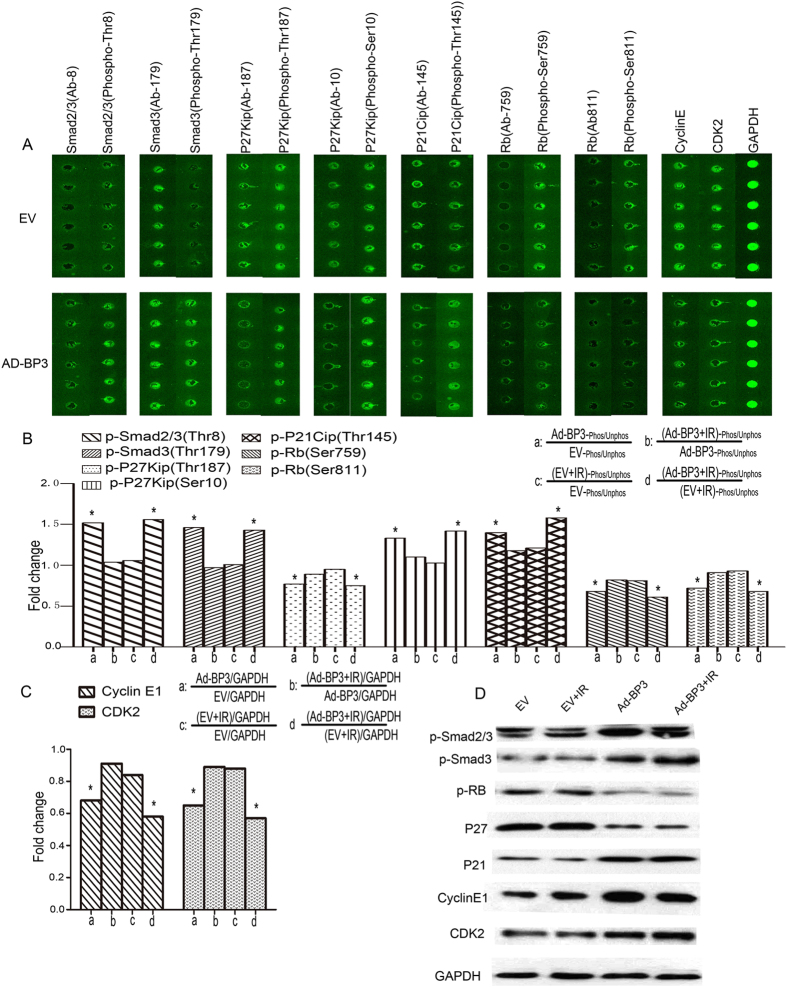
Overexpression of IGFBP-3 regulated the expression and phosphorylation of key cell cycle proteins. (**A**) TE-1 cells infected with EV or Ad-IGFBP3 were treated with or without IR (4 Gy) respectively and were lysed and subjected to an antibody microarray. (**B,C**) Portions of the array illustrate differential expression of cyclin E1 and cyclin-dependent kinase (CDK2) and phosphorylation of Smad2/3, Smad3, and retinoblastoma protein (Rb) in control cells, Ad-IGFBP-3 infected TE-1 cells, control cells after IR treatment and Ad-IGFBP3 infected TE-1 cells after IR treatment .The fold change in cell cycle related proteins was measured. Each panel contains six replicates of a specific antibody–protein reaction. ^*^Compared with control group (*P* < 0.05). (**D**) The expression and phosphorylation of key cell cycle proteins were demonstrated by western blot analysis.

**Figure 7:**
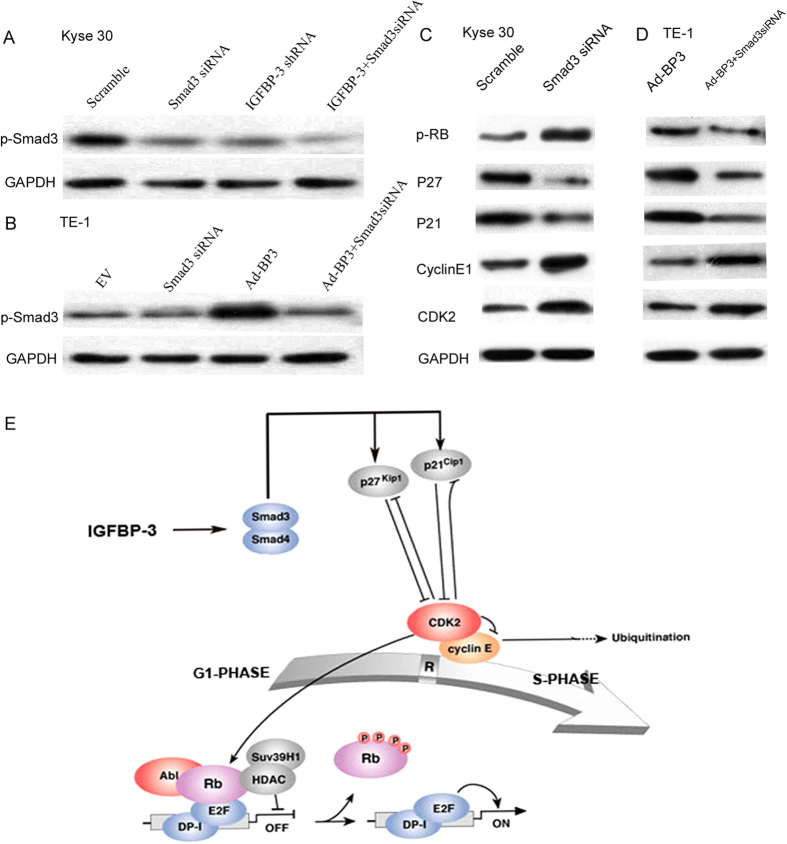
IGFBP-3 promotes esophageal squamous cell carcinoma (ESCC) cell cycle transition from G0/G1 to S phase via Smad3–P27/P21-cyclin E1/cyclin-dependent kinase (CDK2)-phosphorylated retinoblastoma protein (pRb) pathway signaling. (**A,B**) Kyse 30 cells, IGFBP-3-silenced Kyse30 cells, TE-1 cells and was transferred into Smad3-siRNA. Western blot analysis demonstrated the different expression of Smad3 protein. (**C,D**) The expression and phosphorylation of key cell cycle proteins in control cells, Smad3-silenced Kyse 30 cells and Smad3-silenced Ad-IGFBP-3-TE-1 cells were demonstrated by western blot analysis. (**E**) IGFBP-3 phosphorylates Smad3 and induces expression of the CDK inhibitors P21 and P27. These CDK inhibitors facilitate G1 cell cycle arrest by inhibiting cyclin E-mediated CDK2 phosphorylation of Rb. Hyperphosphorylated Rb separates from the E2F transcription factor and releases it to promote the expression of genes that drive cells through G1 into the S phase.

To investigate the molecular basis of these effects of IGFBP-3, we conducted a large scale proteomic screen using antibody arrays to identify proteins that were differentially regulated in Kyse30-Vector cells, Kyse30-IGFBP-3shRNA cells before and after IR. Full Moon Biosystems arrays contain antibodies against phospho and total proteins, including almost 1300 proteins in more than 30 different regulatory pathways. With this array, we discovered decreased phosphorylation of Smad2/3 and Smad3 and increased phosphorylation of Rb by using the formula shown in Fig. 5A,B. As shown in Fig. 5B, in Kyse30-IGFBP-3shRNA cells, Smad2/3 (phospho-Thr8) and Smad3 (phospho-Thr179) were decreased 0.63- and 0.78- fold, respectively, compared with Kyse 30- vector cells Rb (phospho-Ser795 and -Ser811) were increased 1.39- and 1.34-fold, respectively. As shown in Fig. 5C, the antibody assay results indicate that phosphorylation of P27Kip (phospho-Ser10) and P21Cip (Thr145) was decreased 0.73- and 0.75- fold. Phosphorylation of P27Kip (phospho-Thr187) and expression of cyclin E1 and CDK2 were increased 1.32-, 1.45-, and 1.39-fold, respectively. There were no significant changes in Kyse30-vector cells and Kyse30-IGFBP-3shRNA cells after IR. The expression and phosphorylation of key cell cycle proteins was confirmed by Western blot analysis (Fig. 5D). The antibody arrays results shown in Fig. 6 was similar in TE-1-Ad-IGFBP-3 cells and TE-1-vector cells regardless of IR treatment. Furthermore, Kyse 30-IGFBP-3shRNA cells and TE-1-Ad-IGFBP-3 cells was transferred into smad3siRNA or control siRNA. Western blot analysis demonstrated the expression of p-Smad3 protein was known down significantly by Smad3-siRNA. (Fig. 7A,B). We found that after p-Smad3 knockdown, a substantial drop expression of p-RB, P27, P21was displayed but a rise on cyclin E, CDK2 in ESCC cells compared with the control (Fig. 7C,D). As shown in Fig. 7E, it suggested that IGFBP-3 promoted esophageal squamous cell carcinoma (ESCC) cell cycle transition from G0/G1 to S phase via Smad3-P27/P21-cyclin E1/cyclin-dependent kinase (CDK2)–phosphorylated retinoblastoma protein (pRb) pathway signaling.

## Discussion

Radiotherapy is now a major component of the treatment of locally advanced ESCC and strategies that enhance the radiosensitivity of these tumors without causing further toxicity are needed. It has been demonstrated that the EGFR mediates the sensitivity of cancer cells to various cytotoxic treatments, including IR, at least partly through cell cycle phase redistribution^19^,^20^. A previous study reported that a novel molecule downstream of the EGFR in primary and immortalized human esophageal epithelial cells IGFBP-3 was suppressed by the activation of EGF-induced EGFRs in ESCC cell lines^17^,^21^,^22^. There is evidence that levels of IGFBP-3 in EGFR-overexpressing ESCC cells are responsible, for the increased radiosensitivity conferred by nimotuzumab, which improved the radiosensitivity of ESCC cells with high EGFR expression^17^. Although, in another study, Ashida and his colleagues showed that IGFBP-3 overexpression was associated with short-term survivors by definitive chemoradiotherapy in ESCC patients^23^. This fact is adverse to the conclusion of our work. However, in most of publications, authors have made a similar conclusion to this study^10^,^24^,^25^,^26^. Taking these findings together, IGFBP-3 has been suggested to be a key mediator of radiation-induced apoptosis that could act as a radiosensitizer in ESCC, facilitating the development of individualized cancer management.

IGFBP-3 regulates cell proliferation, has proapoptotic effects, and reduces tumor growth through both IGF-dependent and -independent mechanisms^17^. In addition, accumulating evidence shows that the antiproliferative effects of IGFBP-3 may be mediated via activation of caspase-dependent apoptosis. It has been shown that the expression and activity of both proapoptotic and antiapoptotic members of the Bcl-2 family are directly affected by IGFBP-3^27^. In the present study, we investigated the effect of IGFBP-3 on ESCC cell response to IR and the underling mechanisms *in vitro*. We first determined IGFBP-3 levels in five ESCC cell lines. The IGFBP-3 expression of Kyse30 cells was known down by IGFBP-3shRNA. Ad-IGFBP-3 was used to upregulate the expression of IGFBP-3 in TE-1cells. MTT and colony formation assays demonstrated that downregulation of IGFBP-3 increased cell viability and proliferation under IR. Caspase-3 activity and the protein expression ratio of Bax/Bcl-2, which are crucial for the induction of apoptosis, also indicated that IGFBP-3 silencing inhibits apoptosis in ESCC cell lines, before and after IR. Collectively, these data suggest that reduced expression of IGFBP-3 confers resistance to the cell killing effects of IR, whereas upregulation of IGFBP-3 have the reverse effect on ESCC.

Numerous molecules play an important role in regulating cell cycle progression. Increasing evidence suggests that IGFBP-3 is one such molecule. Wu *et al*. reported that IGFBP-3 induced apoptosis through induction of the cell cycle at the G1/S phase, with decreased cyclin E1 expression and increased P21 expression in 786-O, A549, and MCF-7 cells^28^,^29^. Peng *et al*. reported that IGFBP-3 may contribute to androgen-induced antiproliferative effects in LNCaP human prostate cancer cells through increasing CDK1, P21, and P27, leading to G1 arrest^30^. Another study found that IGFBP-3 arrested the cell cycle at G1/S in several cancer cell lines. O’Han *et al*. reported that knockdown of endogenous IGFBP-3 in Hs578T cells stimulated DNA synthesis and promoted cell cycle progression from G0/G1 to S phase and cell growth^31^. In the present study, we confirmed that downregulation of IGFBP-3 expression in Kyse30 cells resulted in a reduction in the percentage of cells in G0/G1 and an increase in S phase. On the contrary, upregulation of IGFBP-3 expression in TE-1 cells enhanced G1/S phase arrest and it might account for radiosensitivity on ESCC. An EdU assay demonstrated that the proportion of cells undergoing DNA synthesis was increased in IGFBP-3-silenced ESCC cell lines. These findings indicate that, in ESCCs with underexpression of IGFBP-3, dividing tumor cells seem to be abundant. Therefore, such ESCC subtype may show radiation or chemoradiation resistance.

Interestingly, we further observe that IGFBP-3 had favorable impact on the tumorigenicity of ESCC cells in nude mice by using an *in vivo* imaging system (IVIS) to monitor tumor growth treated with IR. We confirmed that inhibition of IGFBP-3 suppressed the ESCC killing effect of IR. Upregulation of IGFBP-3 expression strongly reduced tumor growth and improved radiosensitivity *in vivo*. Tumors excised from treated mice which displayed high expression of IGFBP-3had significantly lower levels of the proliferative marker PCNA and higher levers of cell apoptotic index, indicating that IGFBP-3 exerted an anti-proliferative and apoptotic activity *in vivo*. To investigate the mechanism of the effect of IGFBP-3 on the cell cycle, we measured protein phosphorylation using an antibody array containing 1300 targets^32^,^33^. Among cell cycle related proteins, significantly decreased phosphorylation was detected at the Thr8 site of Smad2/3 and at the Try8, Try179, Ser204, and Ser425 sites of Smad3 (data for Smad3 [phospho-Thr8, -Ser204, and -Ser425] not shown). Furthermore, Rb (phospho-Ser780, -Ser795, -Ser807, and -Ser811) were all significantly increased (data for Rb [phospho-Ser780 and -Ser807] not shown). These data imply that the IGFBP-3-promoted G0/G1 to S phase transition might occur though the Smad/Rb pathway.

It is well known that regulation of the cell cycle depends on the cooperative interaction of many key proteins to overcome rate-limiting steps including the G1/S restriction point^34^. CDKs promote cell cycle progression by phosphorylating substrates that control DNA replication, transcription, and mitosis^35^. Cyclin E triggers S phase entry. Overexpression of cyclin E decreases cell size, reduces the need for growth factors, and reaccelerates the G1 phase. Cyclin E has been shown to exert its growth promoting function by binding and activating its catalytic partner CDK2^36^,^37^,^38^. The cyclin E/CDK2 complex phosphorylates several cellular proteins, including Rb, enabling entry into and progression though the S phase. CDK activity is suppressed in part by CDK inhibitors such as the CIP/KIP family (P21waf1/cip1, P27kip1, and P57kip2). It is well established that P21 and P27 have multiple functions in addition to causing G1 arrest by inhibiting cyclin/CDK complex activity, inhibiting or mediating apoptotic signaling pathways in various cancer cell lines. P21 and P27 are negatively correlated with cell proliferation and CDK activity. P27 is also a substrate for phosphorylation at Thr187 by two major cyclin/CDK complexes (cyclin B1/CDK1 and cyclin A or E/CDK2), the strongest determinant of P27 protein instability. Phosphorylation of P27kip at Thr187 by the cyclin E/CDK2 complex triggers P27kip binding to Skp2, a member of the F-box family of proteins that correlates with the Skp1/Cull/Fox protein ubiquitin ligase complex, possibly contributing to P27 ubiquitination and subsequent degradation by the proteasome^39^,^40^. Thus, P21 and P27 are degraded in G1 and G1/S phase. Increasing evidence suggests that phosphorylated Smad3 together with transcription cofactors induces expression of P21 and P27^41^. These CDK inhibitors facilitate G1 arrest by inhibiting cyclin E-mediated CDK2 phosphorylation of Rb^42^. Hyperphosphorylated Rb separates from the E2F transcription factor and releases it to promote the expression of genes that drive cells through G1 into S phase^43^.

Our observations indicate that phosphorylation of P27kip (phospho-Thr187) was upregulated and that of P27kip (phospho-Ser10) is decreased, which means that P27 was downregulated compared with controls when IGFBP-3 protein was knocked down in Kyse30 cells. We also found that phosphorylation of P21cip (phospho-Thr145) was increased in IGFBP-3-silenced cells. Our antibody assay and western blotting results show that expression of P21 and P27 was decreased and that of cyclin E and CDK2 was increased. Upregulation of IGFBP-3 has the reverse effect in TE-1-AdBP-3 cells compared with control. In addition, we found that downregulated the expression of smad3 will also contribute to interrupt these pathways. These results suggest that the IGFBP-3-induced G0/G1 to S phase transition might occur though the Smad3-P27/P21-cyclin E1/CDK2-pRb pathways.

Cell cycle phase determines a cell’s relative radiosensitivity, with cells being most radiosensitive in G2/M, less sensitive in G1, and least sensitive during the latter part of the S phase. G1/S phase arrest is consistently associated with increased radiosensitivity because the cells are in a relatively sensitive phase of the cell cycle and fail to enter the most IR resistant phase^44^. Downregulation of IGFBP-3 may interfere with the G0/G1 to S phase transition, leading to radioresistance^43^. In other words, increased levels of IGFBP-3 have downstream effects that lead to cell accumulation in G1, leading to radiosensitization.

In conclusion, knockdown of IGFBP-3 confers resistance to the cell killing effects of IR on ESCC *in vitro* and *in vivo*. Downregulation of IGFBP-3 promoting ESCC cell cycle transition from G0/G1 to S phase. Upregulation of IGFBP-3 accounted for G0/S phase arrest might be the main cause of the IR radiosensitivity. The Smad3-P27/P21-cyclin E1/CDK2-pRb pathways might contribute to this progression. Identification of IR responsive and non-responsive tumors will be beneficial in providing a framework for future individualized therapy.

## Methods

### Ethics statement

All experimental methods were carried out in accordance with the approved guidelines. Protocols were approved by the Institutional Animal Care and Use Committee of Sun Yat-Sen University Cancer Center, China.

### Cell lines and reagents

The human ESCC cell lines Kyse410, Kyse140, Kyse30, EC109, and TE-1 were obtained from the cell bank of the Chinese Academy of the Sciences (Shanghai, China). All cell lines were cultured in RPMI 1640 medium (Invitrogen, Carlsbad, CA) supplemented with 10% FBS (Bioind, Kibbut Beit, Israel) in a humidified 5% CO_2_ atmosphere at 37 °C.

### Construction of the recombinant lentiviral vector

The IGFBP-3 expression construct was generated by subcloning the PCR-amplified human IGFBP-3 coding sequence into the pAd retroviral vector. The construction of the IGFBP-3 short hairpin RNA (shRNA) lentiviral expression vector and retroviral production and infection have been described previously^45^. Based on their baseline expression of IGFBP-3, ESCC cells were transduced with either IGFBP-3-shRNA or Ad-BP3. Kyse 30 cells showed high expression of IGFBP-3 and were infected with retroviruses carrying IGFBP-3shRNA. The TE-1 cells showed had low expression of IGFBP-3 and were infected with Ad-BP3. The sequence for the IGFBP-3shRNA was r (5′-GGCTTCTGCTGGTGTGTGGATAAGTATGG-3′). These cells were subsequently subjected to the following experiments.

### Small interfering RNA (siRNA) transfection

Cells were plated in six-well dishes at a density of 5 × 10^4^ cells/well and allowed to attach overnight. Transfection was performed according to the manufacturer’s instructions using Lipofectamine™ 2000 (Invitrogen, Carlsbad, CA), diluting the siRNA to a final concentration of 50 nM with Opti-MEM® reduced serum medium (Invitrogen)^46^. Cells were typically harvested 72 h post-transfection in 100 μl lysis buffer and expression knockdown was confirmed by Western blot analysis. The sequence for the anti-Smad3 siRNA was r (5′-AAUGGUGCGAGAAGGCGGUCAdTdT-3′)^47^.

### MTT proliferation assay

Details may be found in the Supplementary materials and methods.

### Colony forming assay

Details may be found in the Supplementary materials and methods.

### Propidium iodide staining and flow cytometry

Details may be found in the Supplementary materials and methods.

### Western blot analysis

This procedure has been described previously^48^. The cells were lysed using a 50 nM Tris (pH 7.5), 150 mM Nacl, and 0.5% NP-40 solution on ice. Fifty micrograms of total protein from each sample was resolved on a 12% bis–tris gel with MOPs running buffer and transferred to nitrocellulose membranes. The blots were probed with various antibodies, including anti-IGFBP3, anti-p-RB, anti-Bcl-2, anti-Caspase-3 (Abcam), anti-Bax, anti-p-Smad3, anti-p-Smad2/3, anti-P27, anti-P21, anti-CDK2, anti-Cyclin E1, and anti-p-Chk1 (Ser345) (Cell Signaling Technology).

### 5-Ethynyl-2-deoxyuridine (EdU) assay

The effect of IGFBP-3 on cell proliferation was determined by EdU incorporation assay. Briefly, Kyse30-IGFBP-3shRNA cells, Kyse30-vector cells, TE-1-AdBP3 cells, TE-1-vector cells were seeded in 96-well culture plates. All cells were treated with 50 μmol/l of EdU for 2 h at 37 °C. After fixing with 4% paraformaldehyde for 15 min, the cells were treated with 0.5% Triton X-100 for 20 min and rinsed with PBS three times. The cells were then exposed to 100 μl of 1 × Apollo^®^ reaction cocktail for 30 min and incubated for 30 min with 5 μg/ml of Hoechst 33342 to stain the nuclei. Images were captured using a fluorescence microscope (Olympus, Tokyo, Japan).

### Generation of bioluminescent ESCC cell lines

Kyse30-IGFBP-3shRNA cells, Kyse30-vector cells, TE-1-AdBP3 cells, TE-1-vector cells were infected with ready-to-use lentiviruses containing a firefly luciferase 2 (luc2) reporter gene and a puromycin resistant gene (Lenti-fire; *In Vivo* Imaging Solutions) in the presence of 6 μg/ml of polybrene (Sigma-Aldrich), followed by screening with puromycin (5 μg/ml) (Invitrogen). The selected cells were analyzed for their luciferase activity by *in vitro* monitoring of bioluminescence (GloMax 20/20 Single Tube Luminometer; Promega). Isolated clones were maintained in complete medium supplemented with 3 μg/ml of puromycin.

### *In vivo* tumor growth assay

6-week-old athymic nude mice were used for *in vivo* experiments. The cells treated with luc2 were injected onto the lateral aspect of the rear leg. Mice were anesthetized with Chloral hydrate and bioluminescent images were measured once a week using an IVIS Spectrum (Xenogen IVIS 100; Caliper). When tumors had grown to a volume of 200 mm^3^, mice were randomized into four groups (eight mice per group): Scramble; IGFBP-3shRNA; Scramble + IR; and IGFBP-3shRNA + IR (IR = ionizing radiation). In the Scramble + IR and IGFBP-3shRNA + IR groups, 6 Gy was delivered to animals restrained in custom lead jigs for localized IR treatment. Tumor progression was monitored once a week by IVIS. Tumors diameters were measured with calipers every 4 days, and tumors volumes were calculated using the formula (width^2^ × length/2).

### Terminal deoxynucleotidyl transferase-mediated dUTP labeling (TUNEL) assay

Details may be found in the Supplementary materials and methods.

### Cell cycle antibody array

An antibody array on total lysates obtained from Kyse30-IGFBP-3shRNA cells, Kyse30-vector cells, TE-1-AdBP3 cells, TE-1-vector cells was performed by Full Moon BioSystems, Inc. (Sunnyvale, CA). This assay is based on the incubation of biotin labeled total cell lysates with array slides on which antibodies are immobilized and the detection of biotin labeled protein–antibody complexes by Cy3-streptavidin.

### Statistical analysis

Details may be found in the Supplementary materials and methods.

## Additional Information

**How to cite this article**: Luo, L.-L. *et al*. Insulin-like growth factor binding protein-3 is a new predictor of radiosensitivity on esophageal squamous cell carcinoma. *Sci. Rep*. **5**, 17336; doi: 10.1038/srep17336 (2015).

## Supplementary Material

Supplementary Information
