# Supplementary material for: Insulin-like growth factor binding protein-3 is a new predictor of radiosensitivity on esophageal squamous cell carcinoma
Source: Sci Rep. 2015 Dec 16;5:17336. doi: 10.1038/srep17336 (PMC4680797; doi:10.1038/srep17336)

Insulin-like growth factor binding protein-3 is a new predictor of radiosensitivity on esophageal squamous cell carcinoma

Li-Ling Luo[1,2,5#]; Lei Zhao[1,2,5#]; Ying-Xue Wang[3#]; Xiao-Peng Tian[2,5]; Mian Xi[1,2,5]; Jing-Xian Shen[2,4,5]; Li-Ru He[1,2,5]; Qiao-Qiao Li[1,2,5]; Shi-Liang Liu[1,2,5]; Peng Zhang[1,2,5]; Dan Xie[2,5]; Meng-Zhong Liu [1,2,5]*

# Li-Ling Luo, Lei Zhao and Ying-Xue Wang contributed equally to this work.

Supplementary Methods

Methods

MTT proliferation assay

The effect of IGFBP-3 on ESCC cell proliferation was assessed by MTT assay in six replicates. Briefly, Kyse30-IGFBP-3shRNA cells, Kyse30-vector cells, TE-1-AdBP3 cells, TE-1-vector cells were seeded in 96-well plates at a density of 8×10^3 cells per well. Treated cells were exposed to different doses of radiation (2, 4, 6, 8, or 10 Gy). Absorbance at 570 nm was determined on a plate reader (Bio-Rad Laboratories, Hercules, CA). Inhibition of cell growth was measured as the percentage of viable cells relative to the controls and calculated as follows: percentage of viable cells = 100% × ODT/ODC, where ODT is the average optical density (OD) of the treated samples and ODC is the average OD of the control samples.

Colony forming assay

The cytotoxic effects of IGFBP-3 on ESCC cells were investigated according to established clonogenic

assay methods. In a six-well plate, about 300 Kyse30-IGFBP-3-shRNA cells, Kyse30-vector cells, TE-1-AdBP-3 cells and TE-1-vector cells were exposed to 2, 4, 6, 8, or 10 Gy of radiation for 48 h in medium supplemented with 1% fetal bovine serum. After 12 days of incubation in complete culture medium, the colonies were stained with crystal violet after fixation with formaldehyde and counted manually. The plating efficiency (PE) and survival fraction (SF) were calculated as follows: PE=(colony number/inoculating cell number) ×100%. SF=PE (tested group)/PE (0-Gy group) ×100%. A dose-survival curve was obtained for each experiment and used for calculating several survival parameters. Parallel samples were set at each radiation dosage. The cell-survival curve was plotted with SigmaPlot 12.5 software (San Jose, CA, USA), using the equation: $SF=1-(1-e\text{-}D/D0)^N$. The multi-target, single-hit model was applied to calculate the cellular radiosensitivity (mean lethal dose, D0), the capacity for sublethal damage repair (quasithreshold dose, Dq), and the extrapolation number (N).

Propidium iodide staining and flow cytometry

Cell cycle progression was determined by flow cytometry. Kyse30-IGFBP-3-shRNA cells, Kyse30-vector cells, TE-1-AdBP-3 cells and TE-1-vector cells were seeded in a 24-well plate at a density of 5×10^4 cells per well. Each well was trypsinized and the cells were fixed with 70% ethanol followed by staining with propidium iodide. The cellular DNA content of each sample was determined by flow cytometry (Becton Dickinson, San Jose, CA). All experiments were performed in triplicate.

Terminal deoxynucleotidyl transferase-mediated dUTP labeling (TUNEL) assay

Apoptotic cells in serial sections of xenograft tumors were determined by TUNEL assay using an In

Situ Cell Death Detection Kit POD (Roche Diagnostics, Mannheim, Germany), which quantitatively determines DNA fragmentation visualized with 3,3'-diaminobenzidine tetrahydrochloride, according to the manufacturer's instructions. The presence of clear nuclear staining (TUNEL positive, brown color) indicated apoptotic cells. Apoptotic bodies were defined as TUNEL positive, single, relatively large (≥ 4 μm diameter) and roundish bodies present in extra- or intratumoral cells with intense staining. The number of TUNEL positive tumor cell nuclei was counted and the apoptotic index (AI) was determined as the percentage of apoptotic cells in the tumor. A minimum of 500 epithelial cells was counted for each case.

Statistical analysis

All statistical analyses were performed using SPSS standard version 13.0. Data derived from cell line experiments were presented as the mean ± standard error and assessed by Student's *t*-test. P values of < 0.05 were considered significant.

Supplementary Figure Legends

Supplementary Figure 1. The viability of cells expressing different levels of IGFBP-3 was determined after IR by MTT assay.

Figure 1

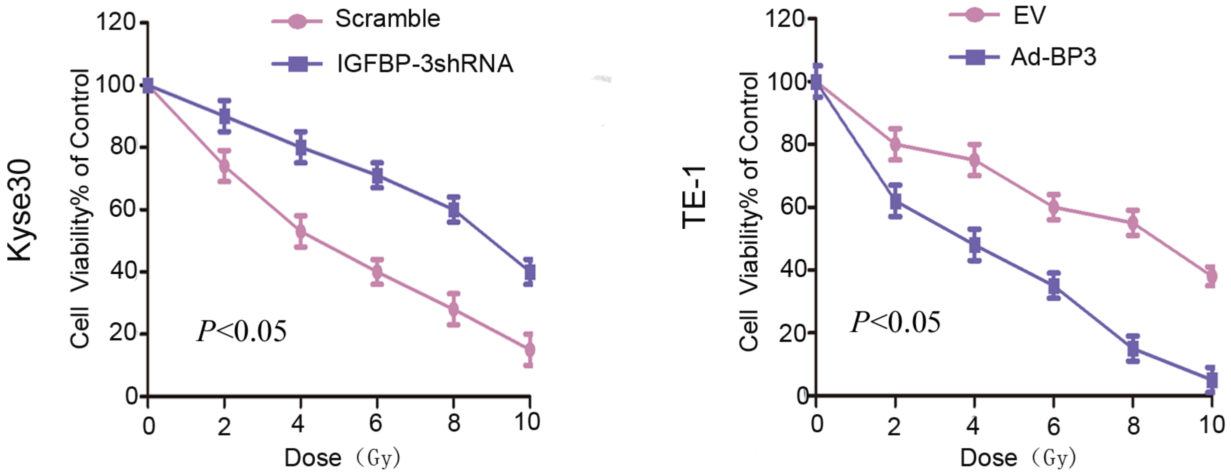

Supplement: Supplementary Information [file srep17336-s1.pdf]
